# The effect of IL-6/Piezo2 on the trigeminal neuropathic pain

**DOI:** 10.18632/aging.202887

**Published:** 2021-04-23

**Authors:** MingXing Liu, Yan Li, Jun Zhong, Lei Xia, NingNing Dou

**Affiliations:** 1 Department of Neurosurgery, Qingdao Municipal Hospital, Qingdao 266000, China; 2 Department of Neurosurgery, XinHua Hospital, The Cranial Nerve Disease Center of Shanghai, Shanghai JiaoTong University School of Medicine, Shanghai 200092, China

**Keywords:** trigeminal neuropathic pain, Piezo2, IL-6, ectopic action potentials, inflammation

## Abstract

The nature of trigeminal neuropathic pain (TN) attacks is regarded as the ignition of ectopic action potentials from the trigeminal root following vascular compression, which seemed to be related to transmembrane proteins and inflammation factors. This study focused on the mechanosensitive channel Piezo2 and cytokine IL-6.

The chronic constriction injury of infraorbital nerve in SD rats was used to establish the TN model. The trigeminal ganglion was then achieved to perform immunocytochemistry studies.

A significant upregulation of Piezo2 and IL-6 was showed in the TN model rats. The Piezo2 positive accounted for 72.3±9.5% in those IL-6 positive neurons. The Piezo2 co-localized with CGRP, IB4 and NF-200 but not with GFAP, which implied that it was expressed in both the C-type and the A-type neurons. After administration of GsMTx4 or anti-rat IL-6 antibody in the TN model, the dynamic allodynia and pinprick hyperalgesia scores as well as the mechanical threshold changed significantly. In the sham-operation rates, with local administration of IL-6, an upregulation of Piezo2 was also exhibited.

Our study demonstrated that the up-regulation of Piezo2 in the pain afferent neurons following trigeminal nerve injury may play a role in the development of the neuralgia. Meanwhile, the expression of Piezo2 may be modulated by inflammatory cytokines, such as IL-6.

## INTRODUCTION

Trigeminal neuralgia (TN) is a commonplace hyperactive cranial nerve disorder due to vascular compression [1–3]. Normally, talking, chewing, washing face, or touching an area of the face merely causes pselaphesia. Why do these ordinary movements or actions turn to neuralgia in TN patients? Researchers have found that after nerve injury even a soft caress may elicit sharp pain (mechanical allodynia). Basically, pain input and tactile lies in IB4+, CGRP+ and NF-200+ neurons respectively [4, 5]. As the mechanosensitive channel is essential to generate an impulse evoked by a gentle touch, could it be possible that this sort of channel proteins emerges from the pain afferent fibers of the trigeminal nerve following some degree of injury?

As a mechanosensitive ion channel, Piezo2 is the first to be identified in trigeminal ganglion (TG) neurons. It was observed that, in Piezo2-deficient mice, dynamic allodynia following nerve injury was absent [6]. This implied the mechanoreceptor could act as an essential mediator of tactile allodynia under inflammatory conditions [7–9]. Previous studies have observed administration of IL-6 neutralizing antibody may significantly decrease the allodynia. Our previous study demonstrated that the serum levels of IL-6 in TN patients were significantly higher than that in healthy volunteers [10, 11].

Hence, the present study aims to verify the hypothesis that the ectopic impulse of allodynia might be generated by Piezo2 mediated by IL-6 following some extent of nerve lesion as the trigeminal nerve has been compressed.

## MATERIALS AND METHODS

### Animals

Adult male Sprague-Dawley rats with weighing 180-220 g were used in the study. The experimental procedures were approved by the Institutional Animal Care and Use Committee of Shanghai JiaoTong University school of medicine. The animals were raised with water and food *ad libitum* in standard laboratory conditions.

### Surgery

We used the chronic constriction injury of infraorbital nerve to establish the trigeminal neuropathic pain model. Without damaging the whiskers, the region between the left eye and whisker pad was shaved under anesthesia (pentobarbital, 50 mg/kg, intraperitoneally). We made a 3-cm long incision caudally to the orbit. After separating the superficial fascia, the infraorbital nerve was exposed and two 4-0 chromic catgut ligatures with a 3-mm length of interval were used to tie the nerve loosely [12, 13] to reduce the nerve diameter noticeably. The same surgical procedure except for the actual nerve ligation was underwent in sham groups [14].

### Innocuous touch assays

### Mechanical threshold

The investigator blinded to the treatment conditions performed the behavioral experiment. With 20 von Frey filaments (North Coast, USA), the mechanical pain threshold was detected. Before the test, the plastic cage environment was used to adapt the rat for at least 1 hour. We stimulated the nerve-injured side whisker pad three times with a 30-seconds interval for six times. Each stimulation began with the lowest force filament and the test was repeated with increasing stiffness filaments until one of the following behaviors occurred twice: escape or attack reactions, brisk head withdrawal or short-lasting facial grooming. According to previous studies, we regarded the successful trigeminal neuropathic pain model as pain threshold was lower than 2 g [21, 22].

### Dynamic allodynia

The 5/0 brush was used to stroke the center of the whisker pad gently. The response was scored as 0 to 3 as previous study showed [15]. The allodynia score was reported as the average scores across three trials per rats.

### Pain assays

### Pinprick hyperalgesia

After the chronic contraction injury, the response to pinprick was scored as 0 to 3 as previous study showed [15]. The hyperalgesia score was reported as the average score across three trials per mouse.

### Drug delivery

In order to deliver drugs to the nerve, we adopted a modified Chacur’s indwelling catheter system [16]. In order to keep patency, we flushed the catheter using a 20-ml saline each day. The drugs were injected through the catheter after four days. To negatively assess the effect of Piezo2 on the innocuous touch and pain, 270 ug/kg GsMTx4 or saline was injected intraperitoneally and the effect was examined every hour. To test the effect of IL-6, the recombinant rat IL-6 (2 ng, Sigma) or goat anti-rat IL-6 antibody (0.04 μg, R&D systems) was administrated in the sham operation or TN model rats once daily for 3 consecutive days [14].

### Specimens

For specimens preparing, with pentobarbital anesthesia (50mg/kg, intraperitoneally), the animals were perfused through the ascending aorta with saline followed by 4% paraformaldehyde in 0.1 M phosphate buffer (4° C, pH 7.2–7.4). We removed the ipsilateral TG after the skull was opened and stored the TG in liquid nitrogen.

### Immunocytochemistry

**Figure 1:**
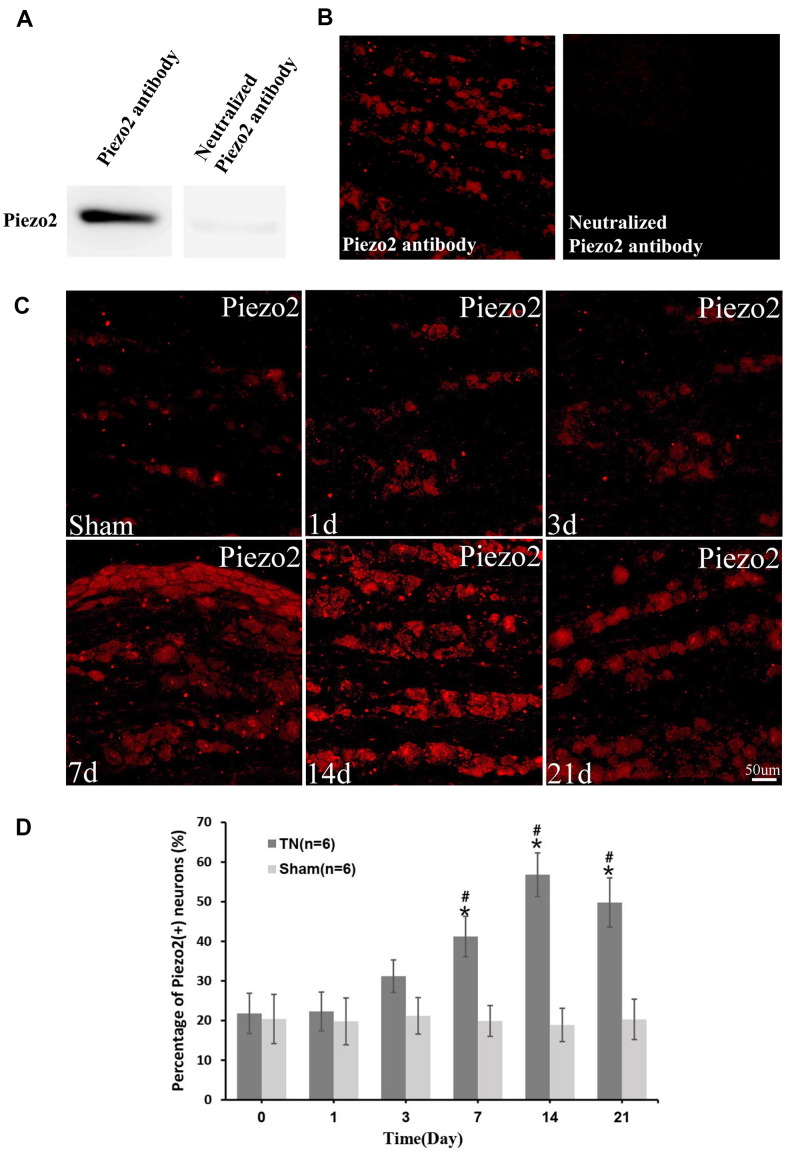
**Immunocytochemistry of Piezo2.** The efficiency of Piezo2 antibody was confirmed by Western blotting and immunofluorescence (**A**, **B**). Immunocytochemistry images show the expression of Piezo2 in sham group and TN group at different time points (**C**). With quantification, the percentage of Piezo2 positive neuron at different time points is displayed, which reaches peak at day 14 (9.2±1.6% in *TN* group *vs.* 2.21±1.1% in *sham* group, *p*<0.05) (**D**). *p<0.05 vs. the sham group; # p<0.05 vs. the bassline.

We used a cryostat to cut the frozen TG into10 μm thick serial coronal sections. All the sections were blocked with 3% donkey serum in 0.3% Triton X-100 for 1 h at room temperature. Then the sections were incubated over two nights at 4° C with goat anti-TNF-α antibody (1:100; Santa), rabbit anti-PIEZO2 antibody (1:100; Abcam). The tissue sections were incubated in Cy3-conjugated donkey anti-rabbit IgG (1:300; Jackson Immuno Research, PA) or Cy3-conjugated donkey anti-goat IgG (1:300, Jackson Immuno Research, USA) for 1 hour at room temperature after the primary antibody incubation. Double immunostaining was performed by incubating with a mixture of anti-Piezo2 and NF200, IB4(FITC conjugated), GFAP or CGRP overnight at 4° C. A mixture of FITC- and Cy3-conjugated secondary antibodies were used to treat all the sections for 1 hour at room temperature. The images of the stained sections were captured with a fluorescence microscope and processed with LEICA IM50 software (Germany). The efficiency of Piezo2 antibody was confirmed by Western blotting and immunofluorescence (Figure 1A, 1B).

### Quantification and statistics

We measured the percentage of positive neurons using LEICA Qwin V3 digital image processing system (Germany). The SAS 8.0 Software was used to analyzed the differences between groups. The data regarding the immunofluorescence and behavioral tests are presented as Mean±SD. The p value less than 0.05 was considered statistically significant.

## RESULTS

### The expression of Piezo2 in TN model rats

The immunocytochemistry displayed a significant Piezo2 upregulation in the TN model rats compared to those with sham surgery (Figure 1C, 1D).

**Figure 2:**
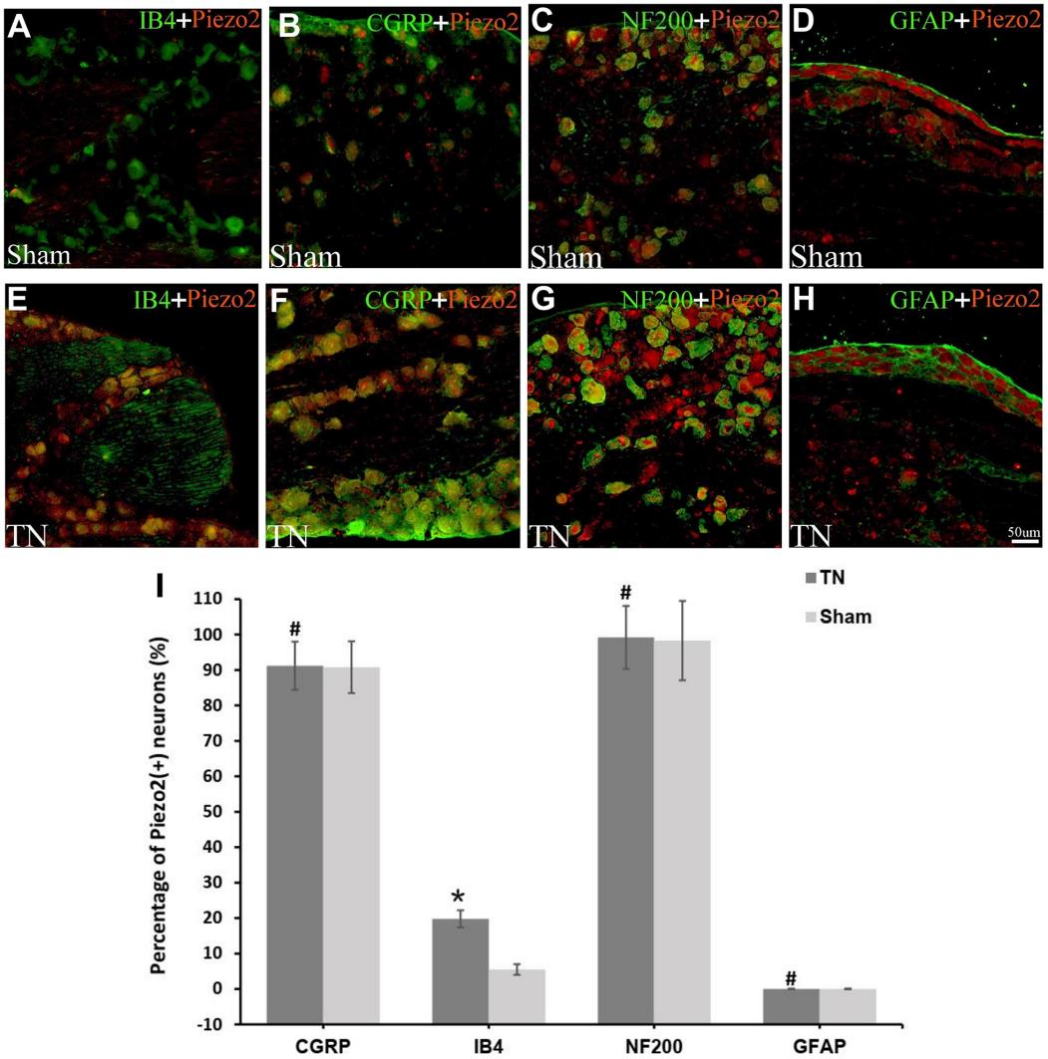
**Double immunofluorescence staining of Piezo2.** Double immunofluorescence staining results are displayed. The red refers to Piezo2, the green refers to IB4 (a marker for C-type neurons), CGRP (a marker for small nociceptive peptidergic neurons), NF-200 (a marker for A-type neurons) or GFAP (a marker for satellite glial cells), and the yellow refers to the colocalization (**A**–**H**). With calculation, the percentage of Piezo2 in IB4 neuron outnumbers those in the other neurons significantly (19.8±2.4% vs. 5.5±1.5%, p<0.05) (**I**).

In order to determine the neuron type whereabouts the Piezo2 expressed, double immunofluorescence staining was performed. The Piezo2 was observed to co-localize with CGRP, IB4 and NF-200 but not with GFAP. The percentages of Piezo2 positive neurons in different cell types were calculated and the result indicated that these in IB4 positive cells increased significantly (Figure 2). It implied that the Piezo2 expressed in both the C-type and the A-type nerve fibers.

### The role of Piezo2 in innocuous touch and pain

**Figure 3:**
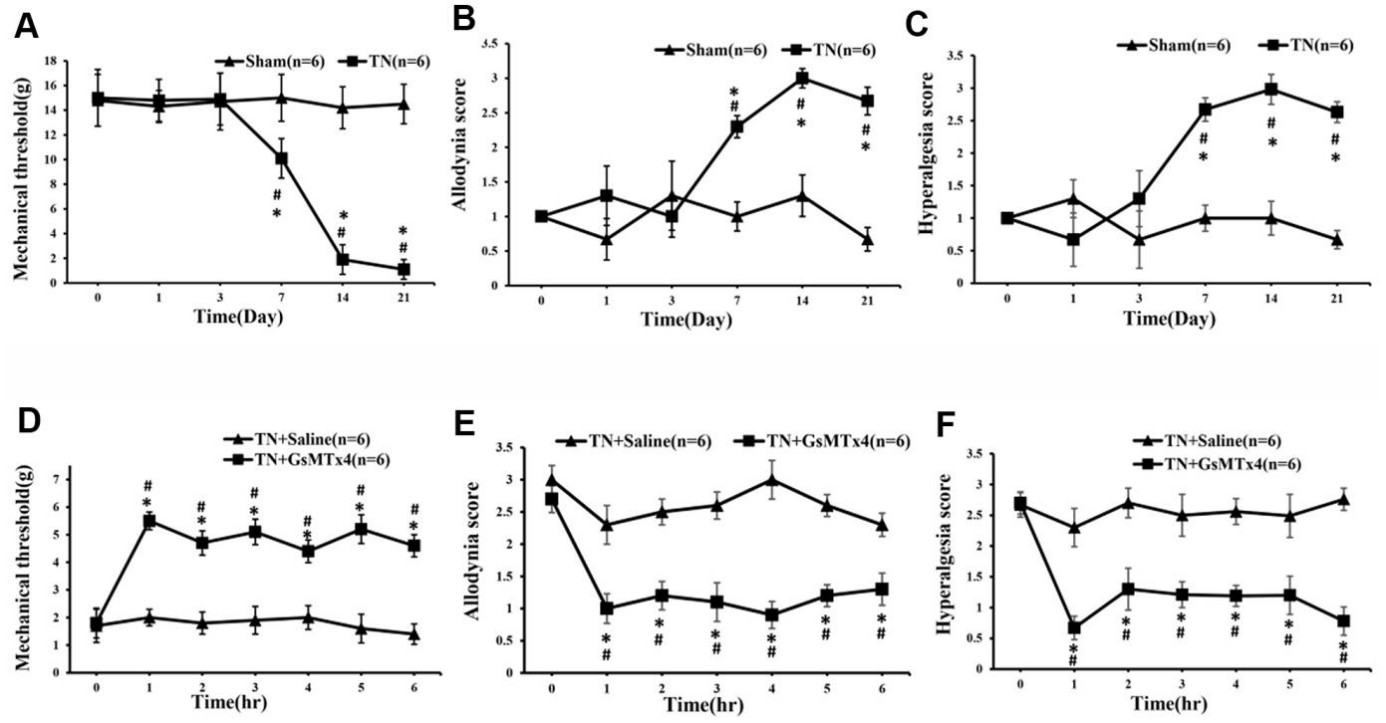
**The alterations of mechanical threshold, dynamic allodynia and pinprick hyperalgesia scores following administration of GsMTx4.** After the contraction injury of the trigeminal nerve, the mechanical threshold began to decrease significantly at day 7, reaching bottom at day 14 and remaining at a low level until the last time point of day 21. Coincidentally, the dynamic allodynia and pinprick hyperalgesia scores changed correspondingly (**A**–**C**). An hour after intraperitoneal administration of GsMTx4 in those TN model rats, the mechanical threshold, dynamic allodynia and pinprick hyperalgesia scores start to change significantly compared to those with saline administration (**D**–**F**).

To assess the effect of Piezo2 on the innocuous touch and pain, 270 ug/kg GsMTx4 (inhibitor of mechano- gated Piezo channels) was injected intraperitoneally in the TN model rats and the effect was examined every hour. It was found that the mechanical threshold increased significantly in the first hour and remained at a high level in the next hours compared to those with intraperitoneal injection of normal saline. Likewise, the dynamic allodynia and pinprick hyperalgesia scores decreased correspondingly (Figure 3).

### The expression of IL-6 in TN model rats

**Figure 4:**
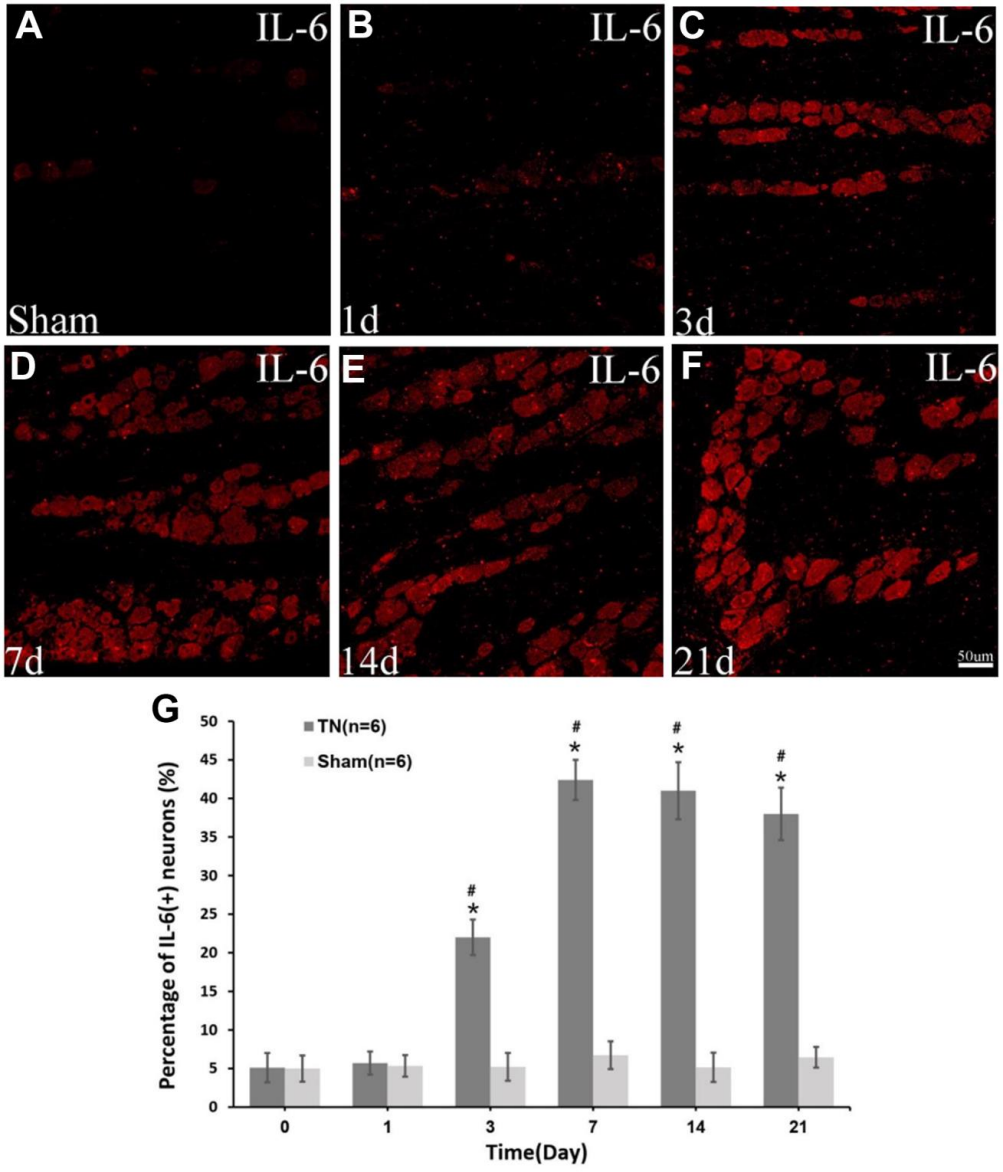
**Immunocytochemistry of IL-6.** Immunocytochemistry images of IL-6 in *sham* group (**A**) and *TN* group at different time points (**B**–**F**) are demonstrated. The percentage of IL-6 (+) neuron increases at day 3 postoperatively, reaching peak at day 7 (42.4±2.6% in *TN* group *vs.* 6.7±1.8% in *sham* group, *p*<0.05) and remaining at a high level up to the last time point of day 21 (**G**).

The immunocytochemistry displayed a significant upregulation of IL-6 in the TN model rats compared to those with sham surgery (Figure 4).

**Figure 5:**
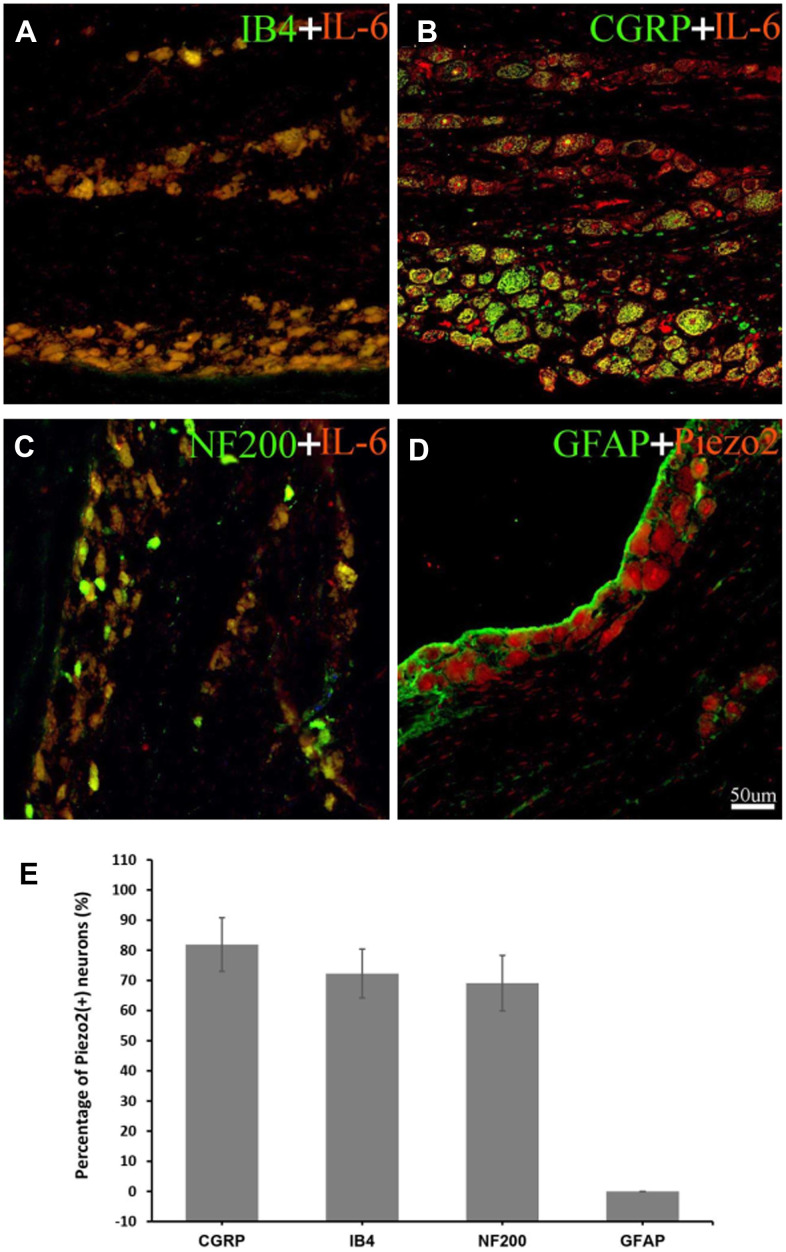
**Double immunofluorescence staining of IL-6.** The double immunofluorescence staining shows IL-6 is co-localized with IB4 (a marker for C-type neurons) (**A**), CGRP (a marker for small nociceptive peptidergic neurons) (**B**) and NF-200 (a marker for A-type neurons) (**C**) but not with GFAP (a marker for satellite glial cells) (**D**). With calculation, the percentage of IL-6 in IB4, CGRP, NF200 neuron was 72.3±8.1%, 81.9±8.9%, 69.1±9.2% respectively (**E**). The red refers to IL-6, the green refers to IB4, CGRP, NF-200 or GFAP, and the yellow refers to the colocalization.

The double immunofluorescence staining showed that IL-6 were co-localized with CGRP, IB4 and NF-200 but not with GFAP, which indicated that the IL-6 expressed predominately in the C-type as well as the A-type nerve fibers (Figure 5).

### The role of IL-6 in Piezo2 expression

**Figure 6:**
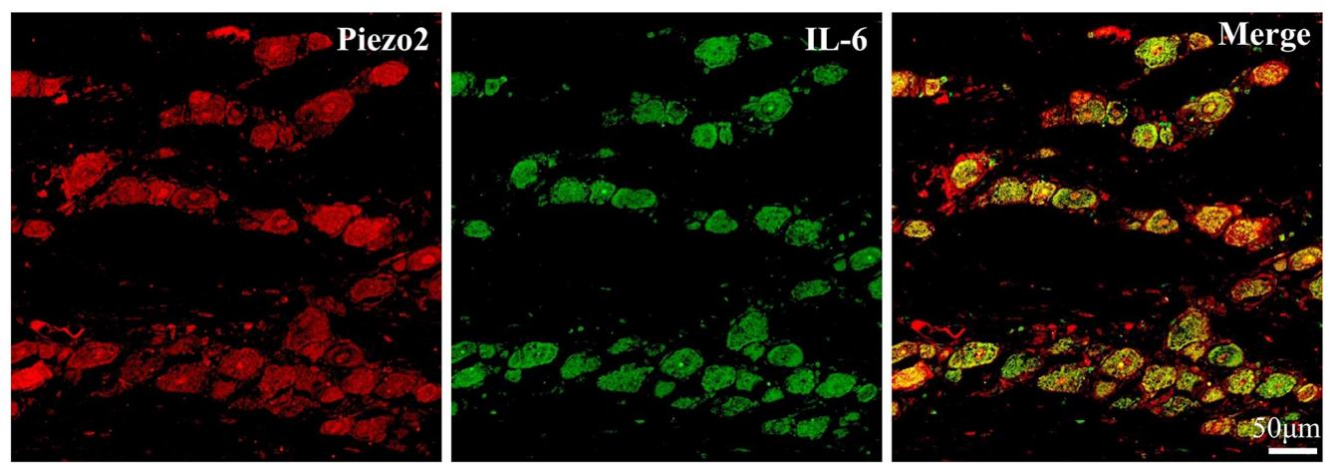
**Double immunofluorescence staining of IL-6 and Piezo2.** The double immunofluorescence staining results exhibit the colocalization (yellow) of IL-6 (Green) and Piezo2 (Red).

To determine the connection between Piezo2 and IL-6 expressions after chronic contraction injury of the trigeminal nerve, double immunofluorescence staining was performed. The results showed the Piezo2 positive accounted for 72.3±9.5% in those IL-6 positive neurons (Figure 6).

**Figure 7:**
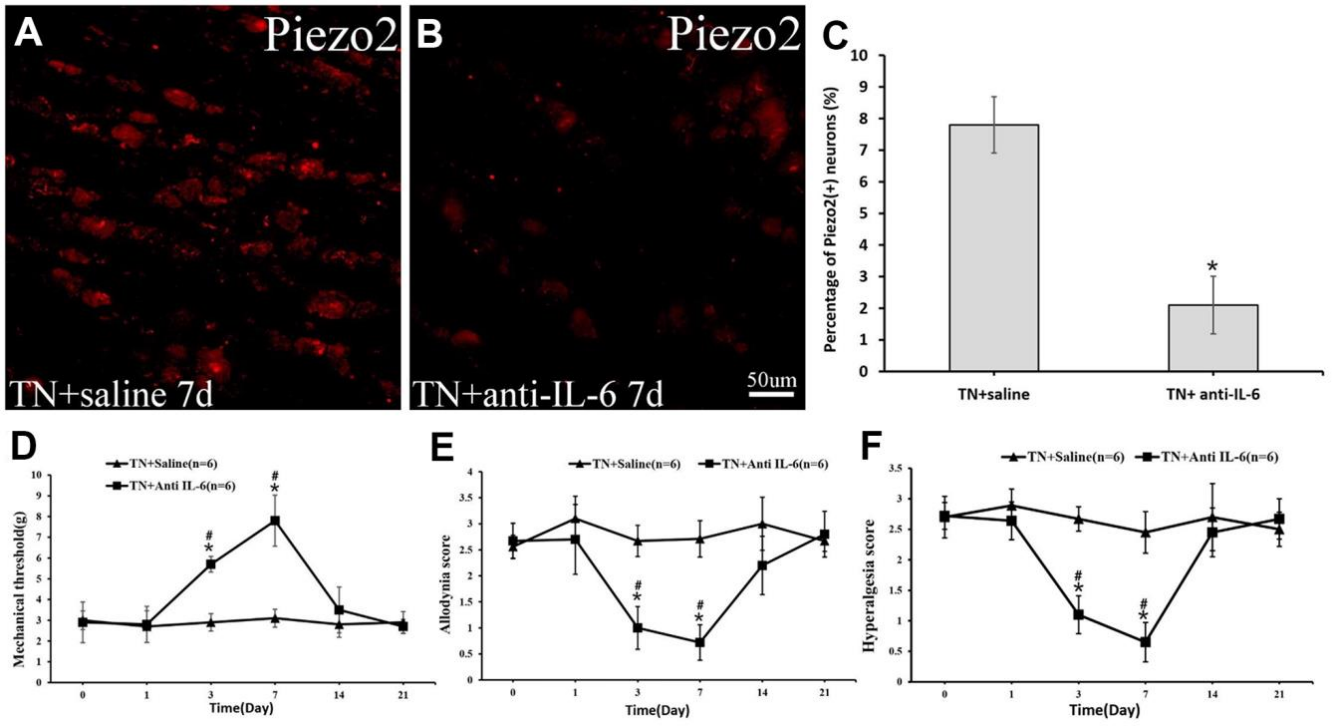
**The Piezo2 expression after anti-IL-6 administration.** Immunocytochemistry imaged show the expression of Pieao2 in *TN+saline* group (**A**) and *TN+anti-IL-6* group (**B**) at day 7, which accounts for 41.2±9.8% vs. 15.1±6.2% (*p*<0.05) (**C**). Three days after intraperitoneally administration of the anti-IL-6 in those trigeminal model rats, significant changes in mechanical threshold (**D**) as well as dynamic allodynia (**E**) and pinprick hyperalgesia scores (**F**) are observed.

After 3 days of administration of goat anti-rat IL-6 antibody in the TN model rats, the mechanical threshold increased significantly and reached peak within a week. Likewise, the dynamic allodynia and pinprick hyperalgesia scores decreased correspondingly. Meanwhile, at day 7, the immunocytochemistry displayed a significant decrease in Piezo2 compared to those with normal-saline-injection TN model rats (Figure 7).

**Figure 8:**
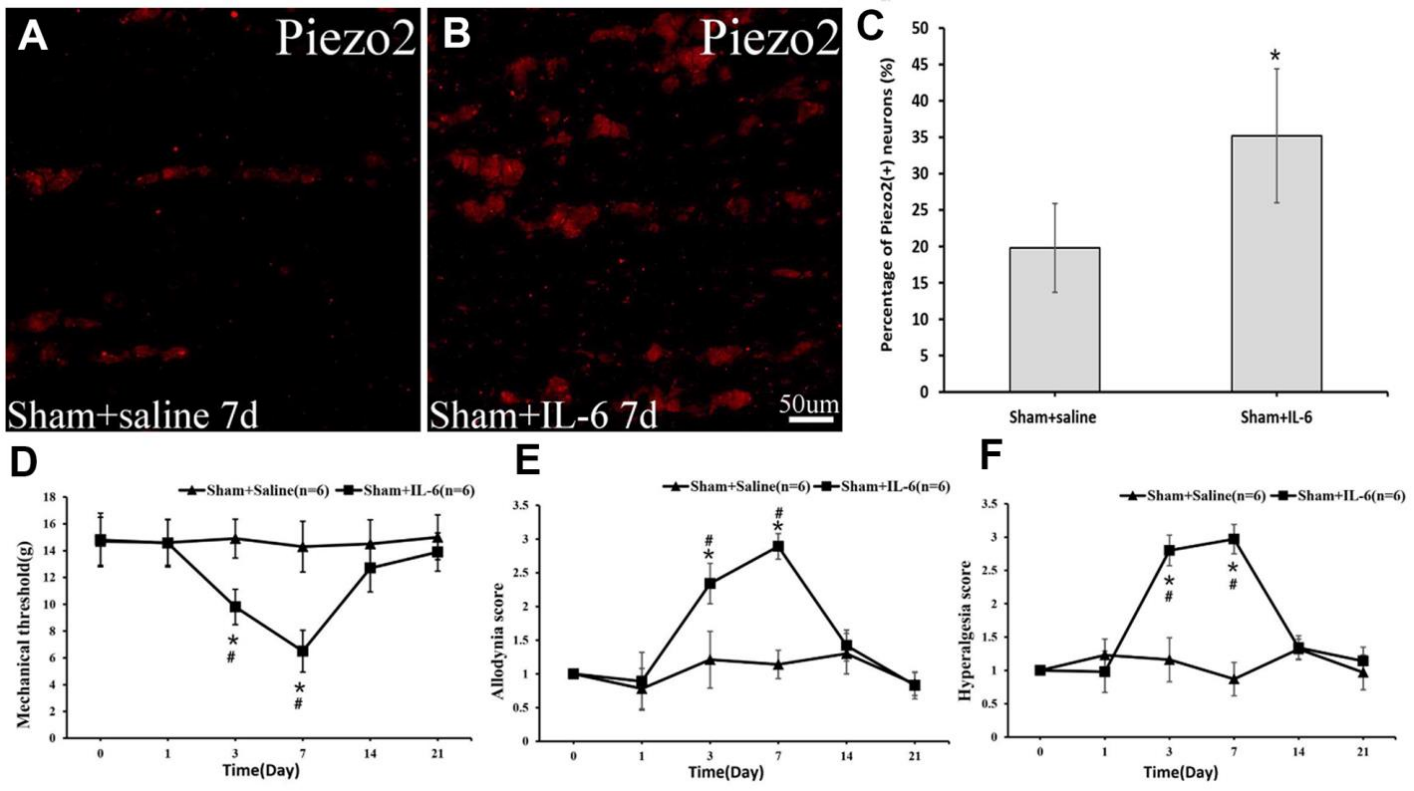
**The Piezo2 expression after IL-6 administration in the sham rats.** Immunocytochemistry images show the expression of Pieao2 in *Sham+saline* (**A**) and *Sham+IL-6* group (**B**) at day 7, which accounts for 19.8±6.1% vs. 35.2±9.2% (*p*<0.05) (**C**). Three days after local administration of recombinant rat IL-6 in those with sham operation, significant changes in mechanical threshold (**D**) as well as dynamic allodynia score (**E**) and pinprick hyperalgesia score (**F**) are observed.

To positively prove the effect of IL-6 on Piezo2 as well as on pain, recombinant rat IL-6 was administrated locally in those with sham operations. Postoperatively, a significant increase in Piezo2 was displayed while the dynamic allodynia and pinprick hyperalgesia scores as well as the mechanical threshold changed significantly (Figure 8).

## DISCUSSION

TN is a common cranial nerve rhizopathy due to the hyperexcitability of the trigeminal nerve. The nature of its attack is believed to be emersions of ectopic action potentials from the trigeminal root following vascular compression [1]. Based on the previous studies, the Na^+^ channels, which need a threshold potential to open, seem to be critical for generation of this ectopic action potential [4, 5, 16]. Given that the episodes of TN are often aroused by mild touch (trigger), we assumed that some sort of mechanosensory channel might be involved. As a Ca^2+^-permeable nonselective cation channel, Piezo has been found to participate in several processes. Recently, the Piezo2 has been already found to express in A-type nerve fibers of trigeminal ganglion for pselaphesia afference [7, 9, 17]. Then, is there any possibility of Piezo2 expression in those allodynia afferent neurons?

### The role of Piezo2 in allodynia

Apart from that in the DRG neurons, recent studies have shown that Piezo2 mediates mechanical transduction in dental primary afferent (DPA) neurons, especially in neurons that contain CGRP. As the CGRP is a biomarker of pain conducting, we consequently inferred that the Piezo2 might play a role in the allodynia of TN [18–20]. Previous study also showed that Piezo2 contributes to nociception by Aδ-mechanoreceptors in noninflamed corneas [21].

In the present study, not only in A- type but also in C-type neurons the Piezo2 expression was observed. It implies that the pain might be conducted by the Piezo2 following the trigeminal nerve injury. This mechanosensitive channel translates those mechanical signals evoked by mild touch on the face into electrical signals. It then drives the resting membrane potential toward depolarization and generates a conductible ectopic action potential [22–24]. Sometimes, this mechanosensor might be also triggered by some extent of vascular compression elicited by the transient alterations of blood pressure or heart rate, which can be well explained the clinical phenomenon that the symptom tends to be aroused by emotion [25].

### The IL-6 effect on Piezo2

Where the premise of ectopic action potential generation seems to be found, how does the Piezo2 arise in the allodynia afferent neurons following nerve lesion? Previous study showed that inflammation plays an important role in the signal pathway leading to development of transmembrane ion channels. Several lines of evidence have also demonstrated that cytokines contribute to the development of neuropathic pain [26–29]. Among all the cytokines, IL-6 o played a pivotal role in the induction of neuropathic pain following nerve injury. Previous studies showed that increased IL-6 may lead to over-expression of sodium channels. It was found that administration of IL-6 produced lasting mechanical allodynia with sodium channel up-regulation in DRG neurons [10, 11]. While there is evidence that Piezo2 is enhanced in sensory neurons by various inflammatory signaling pathways such as Gq-coupled receptors and the cyclic adenosine monophosphate pathway [6].

## CONCLUSIONS

Our study demonstrated that the Piezo2 increase in the IB4 positive neurons following trigeminal nerve injury might play a role in the neuralgia development. Meanwhile, the expression of Piezo2 might be activated by inflammatory cytokines, such as IL-6. Our findings implicated a possibility to cure the trigeminal neuralgia with a noninvasive targeting therapy instead of open-brain surgery in the future as long as we could block the IL-6/Piezo2 pathway.

### Ethics approval and consent to participate

All animal protocols carefully followed Canadian Committee on Animal Care (CCAC) or Association for Assessment and Accreditation of Laboratory Animal Care International (AAALAC) guidelines.
